# Psychosocial Development in Saudi Adolescents With Type 1 Diabetes Mellitus: A Descriptive Comparative Study

**DOI:** 10.7759/cureus.42270

**Published:** 2023-07-21

**Authors:** Abdulaziz Hamzah, Mishary A Alassiri, Al-Shaheen Al-Ghamdi, Saleh Al-Zahrani, Rania Zahid, Waleed Alshehri, Abdullah Alsulami, Nawaf F Halabi, Rabea Baatya, Nawwaf A Almalky, Sami Maghrabi, Ibrahim Nafadi, Abdulrahman Alsubhi, Jehad Halabi

**Affiliations:** 1 College of Medicine, King Saud Bin Abdulaziz University for Health Sciences, Jeddah, SAU; 2 Department of Research, King Abdullah International Medical Research Center, Jeddah, SAU; 3 College of Nursing, Qatar University, Doha, QAT

**Keywords:** adolescent diabetes, diabetes, child and adolescent psychiatry, psychosocial development, diabetes miletus

## Abstract

Background

Diabetes mellitus imposes a significant psychological and social burden on the affected individuals. The impact of type 1 diabetes mellitus (T1DM) on psychosocial development has not been well investigated in the literature. We aim to fill the aforementioned gap by conducting a comparative study to accurately assess the impact of this chronic disease on psychosocial development among adolescents in Saudi Arabia.

Methodology

This structured, phone-based, comparative, and cross-sectional study targets adolescents with T1DM and those without diabetes in Jeddah, Saudi Arabia. Our study utilized a validated instrument psychosocial inventory of ego strengths (PIES), to assess the psychosocial development among the participants. An IRB approval has been granted for this study. The data were analyzed using SPSS. The data collection spanned the duration from November 1, 2020, until June 8, 2021.

Results

A total of 310 individuals were included in the study, 90 of whom were adolescents living with diabetes, and 220 were adolescents not living with diabetes. This study indicates that the individuals with diabetes showed significantly lower development in the Hope and Care subscales compared to the control group. We found no significant correlation between HbA1C levels and scores on the psychosocial development subscales. With regards to comorbidities, adolescents living with T1DM had significantly higher rates than the control group, with asthma being the most frequently reported comorbidity.

Conclusion

This study in Saudi Arabia found that adolescents living with diabetes demonstrated lower scores in Hope and Care subscales compared to adolescents not living with diabetes. It highlights the importance of healthcare professionals monitoring and addressing the psychosocial needs of T1DM patients through a multidisciplinary approach and referral to specialized support services when necessary. Further research and interventions are needed to promote the psychosocial well-being of individuals with T1DM.

## Introduction

Type 1 diabetes mellitus (T1DM) is a significant public health problem affecting nearly 1,110,100 individuals worldwide between the ages of 0 and 19, as reported by the International Diabetes Federation [1]. In Saudi Arabia, between 1990 and 2007, there was a significant surge in the incidence rates of T1DM among children aged 0-14 years [2]. Spanning less than two decades, the incidence doubled from 18.05 cases per 100,000 children during 1990-1998 to 36.99 cases per 100,000 children during 1999-2007. This translates to an average annual increase of 16.8% in the incidence rate. Similar studies have also reported elevated incidence rates of T1DM in Saudi children, ranging from 27.5 to 29 cases per 100,000 individuals. These findings underscore the relatively high incidence of T1DM in Saudi Arabia compared to other countries [2]. T1DM is a prevalent endocrine, metabolic disorder that primarily affects children and adolescents worldwide. It is characterized by the autoimmune destruction of pancreatic β-cells, resulting in a significant deficiency of insulin production. The diagnosis of T1DM imposes a demanding therapy regimen that profoundly impacts the lives of patients and their families. This therapeutic burden exposes them to persistent psychological stress as they navigate the management and daily challenges associated with their condition. Additionally, managing diabetes often requires the active involvement and support of the entire family, which can strain family relationships. Many studies have consistently demonstrated a link between diabetes mellitus and an increased psychological burden, including a higher prevalence of depression [2]. In Saudi Arabia, a few studies have associated T1DM in adolescents with having more psychological problems, depression, and poorer quality of life measures [2,3]. Erik Erikson, a prominent psychologist and psychoanalyst, introduced the influential theory known as Erikson's Stages of Psychosocial Development in the 1950s. This theory expands upon Freud's psychosexual development theory by incorporating social dynamics and extending the concept of psychosocial development into adulthood. It encompasses eight sequential stages of human development, which are influenced by biological, psychological, and social factors throughout an individual's life. This comprehensive bio-psychosocial framework has significantly impacted various fields of study. Erikson's theory encompasses various stages that individuals go through as they grow and face new challenges in childhood, adolescence, and adulthood. Each stage involves two opposing positive/negative psychological tendencies. Successfully resolving these conflicts leads to developing personal strengths and virtues, while unresolved conflicts can hinder healthy development [4].

Erikson's psychosocial theory encompasses eight stages of development. The initial stage is about trust against mistrust, which occurs from birth to eighteen months. In this stage, infants rely primarily on their caregivers. Success in this stage cultivates the virtue of hope; however, failing to develop through this stage will lead to higher levels of fear. The second stage spans from eighteen months to three years old and is known as autonomy against shame and doubt. Children at this age develop a sense of independence and control. Encouragement and support in those aspects will aid in the success of this stage and will develop the virtue of will. Thirdly, during the play age period, the conflict between initiative and guilt, the interaction of children with their peers is essential in this stage, and children, if given the opportunity, will develop a sense of initiative and have confidence in their personal capacity to lead others and make decisions. Success at this stage leads to a sense of purpose. The following three stages of Erikson's social theory include industry as opposed to inferiority, which is the fourth stage, which starts at the age of five and ends at the age of 12. In this stage, children seek the approval of their peers in the form of showcasing their valued skills and gaining pride in their accomplishments. During this age, encouragement leads to a sense of competence, and discouragement leads to inferiority. Competence is the virtue attained from the success of this stage. Next, the fifth stage in Erikson's theory is identity, contrary to role confusion during adolescence, which occurs between 12 and 18 years of age. During this time, adolescents explore their values, beliefs, and roles in order to realize their true essence and establish their own identity. Sexual and occupational identities are two significant aspects of this stage. Fidelity, loyalty, and commitment to oneself are attained from success in this stage. The sixth stage discusses intimacy as opposed to isolation, which is experienced in the age period between 18 and 40 years of age. The focus of this stage is forming intimate and loving relationships. Success in this stage leads to fulfilling connections with others, while failure leads to isolation. Finally, the last two stages span from the age of 40 to more than 65. The seventh stage focuses on generativity in contrast to stagnation. Erikson explains that individuals in this stage strive to make a lasting impact by initiating positive changes that could outlive their personal existence. Success in this stage results in a feeling of usefulness and care, whereas failure is characterized as shallow engagement with the world, leading to a sense of rejection. Lastly, the eighth stage revolves around the conflict between ego integrity and despair. In this stage, an individual might reflect on one's life and either have a feeling of satisfaction and happiness or a deep sense of regret. Wisdom is the final virtue signified in this stage's success [4,5]. This cross-sectional study aims to compare aspects of psychosocial development between adolescents living with diabetes and adolescents not living with diabetes by determining the differently affected psychosocial development factors. Additionally, the study aims to determine whether there is a correlation between HbA1c levels, an indicator of the level of diabetes control, and scores of certain psychosocial development variables. Our study assesses psychosocial development factors in T1DM adolescents in Saudi Arabia using a validated and Arabic-translated questionnaire that assesses psychosocial development based on Erikson's theory [6,7]. This article was previously posted to the Research Square preprint server on November 23, 2022.

## Materials and methods

Study setting

A cross-sectional study was conducted on Saudi adolescents aged 12-18 years, living with T1DM and registered at the King Abdulaziz Medical City, National Guard Health Affairs (NGHA), Western region (KAMC-NGHA-WR). We also reached out to adolescents without T1DM through schools affiliated with Jeddah NGHA and invited them to participate in the study as a comparative (control) sample. The inclusion criteria included age 12-18 years, can independently read and write in Arabic or English, being diagnosed with T1DM for at least 12 months (for the non-control sample), and undergoing diabetes therapy (for the non-control sample). Those having a cognitive impairment or learning disability were excluded from the study. Non-control sample criteria, including diabetes diagnosis, were confirmed through the medical records of NGHA. The control group included adolescents in the same age group as their diabetic peers and non-diabetics. All data were kept confidential. Informed consent was granted prior to participation in the study by the participants or their legal guardians. The Institutional Review Board approved the study at King Abdullah International Medical Research Center (Project #SP20/064/J).

Questionnaire instrument

**Figure 1:**
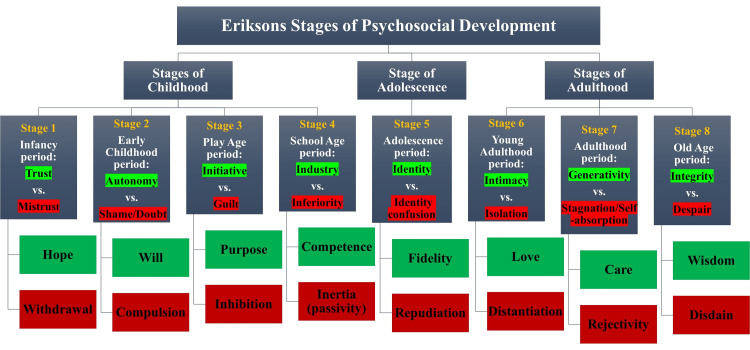
Erikson's stages of psychosocial development. Each stage contains two results, a positive (colored green) and a negative (colored red).

The instrument used in this study was the PIES, a 32-item questionnaire based on Erikson's psychosocial development theory. It consists of eight subscales that correspond to the stages described by Erikson (Figure 1) [4,6,7]. We utilized the validated Arabic-translated version by Alghamdi HA in Saudi Arabia [7]. Participants were given the choice to complete the survey in either English or Arabic. The questionnaire assesses variables related to Erikson's psychosocial development theory, including the ego strengths: Hope, Will, Purpose, Competence, Fidelity, Love, Care, and Wisdom. Each item was scored on a Likert-type scale ranging from 1 (does not describe me well) to 5 (describes me very well), with reversed scoring for items assessing the absence of ego virtue.

In addition to the PIES questionnaire, demographic characteristics were collected, including age, gender, Hb1AC levels provided by the participant or the health record (for the non-control sample), and the presence of any comorbidities.

Data collection

The sampling technique utilized was non-probability convenience sampling. Master lists of phone numbers were requested from the National Guard Hospital in Jeddah, Saudi Arabia, for patients meeting our inclusion criteria. Similarly, master lists of phone numbers were obtained from administrators in the six schools contacted for the control sample. The authors assigned 13 well-trained data collectors, who were instructed to conduct the questionnaire. Data was directly entered into an Excel sheet designed specifically for the study. Data collection spanned from November 1, 2020, until June 8, 2021.

Data analysis

The data were analyzed using SPSS version 21 (IBM Corp., Armonk, NY). Both descriptive and inferential statistics were conducted. A p-value cut-off point of 0.05 at 95% CI was considered statistically significant, while a p-value of 0.01 was considered highly statistically significant. The psychological development of the participants has been assessed using the psychosocial inventory of ego strengths (PIES). Normality tests were performed using the Shapiro-Wilk and the Kolmogorov-Smirnov tests. Furthermore, the comparison between having diabetes among the socio-demographic characteristics and the domains of PIES was conducted using the Chi-square test (categorical variables) and Mann Whitney U-test (continuous variable vs. two categorical variables) or Kruskal Wallis H-test (continuous variable vs. three or more categorical variables). Significant results were then placed in a regression model to determine the independent influence of psychosocial development among patients with T1DM with corresponding odds ratio and 95% CI. The overall PIES score and its domains follow the abnormal distribution. Thus, non-parametric tests were applied. Pearson correlation coefficient was also performed to determine the correlation between the PIES score and its domains along with the HbA1c level.

## Results

**Figure 2:**
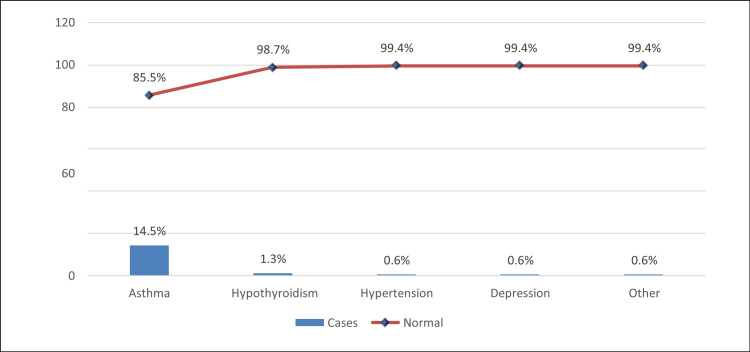
Associated comorbidities.

A total of 310 individuals were recruited, 90 of whom were adolescents living with diabetes, and 220 were adolescents not living with diabetes. The most common age group was 15-17 (61%). Males (50.6%) were slightly more than females (49.4%), and 17.7% had associated comorbidities. The mean value of HbA1c was 11.3 (SD 3.24). The statistical test revealed that T1DM was more common among those with comorbidities (p<0.001). Table 1 describes the demographic characteristics of the participants. It was observed that the most common type of associated comorbidity was asthma (14.5%) and hypothyroidism (1.3%) (Figure 2).

**Table 1:** Demographic data of the participants. § P-value has been calculated using Chi-square test. ** Significant at p<0.05 level.

	Overall N (%) (n=310)	Type 1 Diabetes		
Study Data		Yes N (%) (n=90)	No N (%) (n=220)	P-value §
Age group				
12-14 years	56 (18.1%)	14 (15.6%)	42 (19.1%)	
15-17 years	189 (61.0%)	54 (60.0%)	135 (61.4%)	0.551
18 years	65 (21.0%)	22 (24.4%)	43 (19.5%)	
Gender				
Male	157 (50.6%)	52 (57.8%)	105 (47.7%)	0.108
Female	153 (49.4%)	38 (42.2%)	115 (52.3%)	
Having comorbidities				
Yes	55 (17.7%)	32 (35.6%)	23 (10.5%)	<0.001 **
No	255 (82.3%)	58 (64.4%)	197 (89.5%)	
HbA1c (%) (mean ± SD)	11.3 ± 3.24	11.3 ± 3.24	--	--

Furthermore, the overall total positive and negative scale mean values were 55.9 and 49.5, respectively, while the overall mean PIES score was 105.4. When compared to the patients with T1DM, it was observed that the mean values of Hope (p=0.009) and Care domains (p=0.006) were statistically significantly lower among patients with T1DM. The descriptive statistics of the PIES questionnaire are given in Table 2.

**Table 2:** Inferential statistics of PIES questionnaire comparing diabetics to non-diabetics (n=310). PIES: Psychosocial inventory of ego strengths § P-value has been calculated using Mann-Whitney Z-test. ** Significant at p<0.05 level.

PIES Parameters	Overall mean ± SD	Type 1 Diabetes		Z-test	P-value
		Yes	No		
Hope	7.35 ± 2.35	6.81 ± 2.42	7.57 ± 2.29	2.599	0.009 **
Withdrawal	5.97 ± 2.59	5.79 ± 3.09	6.04 ± 2.37	0.782	0.434
Hope vs Withdrawal	13.3 ± 4.35	12.6 ± 4.95	13.6 ± 4.05	1.743	0.081
Will	6.14 ± 2.27	6.28 ± 2.23	6.08 ± 2.28	0.964	0.335
Compulsion	6.34 ± 2.59	6.37 ± 2.18	6.32 ± 2.74	0.150	0.881
Will vs Compulsion	12.5 ± 4.40	12.6 ± 3.75	12.4 ± 4.65	0.481	0.630
Purpose	6.72 ± 2.04	6.60 ± 1.98	6.77 ± 2.07	0.802	0.423
Inhibition	6.36 ± 2.99	6.10 ± 2.81	6.47 ± 3.06	0.957	0.338
Purpose vs Inhibition	13.1 ± 4.64	12.7 ± 3.97	13.2 ± 4.89	1.233	0.217
Competence	7.18 ± 1.89	6.91 ± 2.08	7.29 ± 1.79	1.578	0.115
Inertia	5.81 ± 2.58	6.18 ± 2.18	5.66 ± 2.71	1.408	0.159
Competence vs Inertia	12.9 ± 3.82	13.1 ± 3.42	12.9 ± 3.98	0.133	0.894
Fidelity	7.66 ± 1.59	7.46 ± 1.77	7.75 ± 1.52	1.084	0.742
Role repudiation	6.29 ± 2.62	6.21 ± 2.92	6.32 ± 2.49	0.822	0.411
Fidelity vs Role repudiation	13.9 ± 3.39	13.7 ± 3.88	14.1 ± 3.17	1.494	0.135
Love	7.02 ± 1.82	7.19 ± 1.89	6.95 ± 1.79	0.110	0.913
Exclusivity	7.31 ± 2.55	7.41 ± 2.31	7.27 ± 2.64	0.717	0.473
Love vs Exclusivity	14.3 ± 3.78	14.6 ± 3.68	14.2 ± 3.83	1.494	0.135
Care	8.18 ± 1.93	7.74 ± 2.01	8.36 ± 1.86	2.742	0.006 **
Rejectivity	5.99 ± 2.72	5.89 ± 2.16	6.03 ± 2.92	0.045	0.964
Care vs Rejectivity	14.2 ± 4.03	13.6 ± 3.44	14.4 ± 4.23	1.627	0.104
Wisdom	5.62 ± 2.36	5.70 ± 2.61	5.59 ± 2.26	0.048	0.962
Disdain	5.46 ± 2.41	5.26 ± 2.89	5.55 ± 2.19	0.962	0.336
Wisdom vs Disdain	11.1 ± 4.02	10.9 ± 4.92	11.1 ± 3.59	0.171	0.864
Total positive scales	55.9 ± 12.5	54.7 ± 12.4	56.3 ± 12.6	0.754	0.451
Total negative scales	49.5 ± 16.9	49.2 ± 16.0	49.7 ± 17.2	0.152	0.879
Overall PIES score	105.4 ± 27.6	103.9 ± 25.9	106.0 ± 28.4	1.068	0.286

Moreover, the correlation between HbA1c among the overall PIES score and its domains did not reach statistical significance (Table 3). 

**Table 3:** Correlation (Pearson-r) between HbA1c R-value and PIES scores (n=310). PIES: Psychosocial inventory of ego strengths.

PIES Parameters	HbA1c R-value	P-value
Hope	0.130	0.222
Withdrawal	0.011	0.920
Will	0.036	0.735
Compulsion	-0.106	0.318
Purpose	-0.073	0.497
Inhibition	-0.033	0.757
Competence	0.022	0.836
Inertia	-0.107	0.317
Fidelity	-0.003	0.976
Role repudiation	-0.077	0.471
Love	0.000	0.997
Exclusivity	-0.171	0.106
Care	-0.014	0.898
Rejectivity	0.017	0.877
Wisdom	-0.057	0.596
Disdain	0.000	0.999
Total positive scales	0.010	0.929
Total negative scales	-0.069	0.517
Overall PIES score	-0.038	0.721

## Discussion

In this study, we aimed to compare aspects of psychosocial development between Saudi adolescents living with T1DM and those not living with diabetes. We also explored the correlation between HbA1c levels, an indicator of diabetes control, and psychosocial development variables. Our findings provide valuable insights into the psychosocial well-being of Saudi adolescents with T1DM and shed light on the potential impact of diabetes on their development. To our knowledge, this study is the first in local literature to investigate the psychosocial development among T1DM individuals. This study makes a valuable contribution to the existing literature focusing on the psychological impact of diabetes by exploring the potential psychological predispositions or underlying factors associated with the development of depression in individuals living with diabetes. The available literature offers various pieces of evidence focusing on the relationship between diabetes and depression. In line with this, a study conducted by Gendelman N et al., published in Diabetes Care, aimed to comprehensively investigate the prevalence of depression among individuals with T1DM compared to those without T1DM. The study included 1,004 participants diagnosed with T1DM and a control group. Both groups completed the Beck Depression Inventory-II (BDI-II), a 21-item self-reported questionnaire specifically designed to assess depressive symptoms in adults and adolescents. The findings of their study revealed a significant difference in the prevalence of depression between the two groups. The prevalence of depression among individuals living with T1DM was significantly higher, with 32.1% of them scoring above the threshold for depression on the BDI-II questionnaire. In contrast, only 16.0% of the control group participants had scores surpassing the established threshold. These results indicate that T1DM may serve as a potential contributing factor for the development of depression in individuals with T1DM compared to their non-diabetic peers [8]. On the other hand, a systematic review conducted by Johnson B et al., published in Diabetic Medicine, analyzed 23 articles examining the relationship between depression and T1DM among children, adolescents, and young adults up to 25 years of age. The review concluded that the current literature lacks conclusive evidence regarding the correlation between diabetes and depression within this particular age group [9]. The findings of our study indicate that adolescents living with diabetes exhibit lower levels of psychosocial development, specifically in the subscales of Hope and Care, compared to adolescents without diabetes. These differences were found to be statistically significant, with p-values of 0.009 for the Hope subscale and 0.006 for the Care subscale. These findings align with Erikson's psychosocial theory, which suggests that the inability to successfully resolve the theme of Hope can lead to issues related to disconnection, such as feelings of abandonment, mistrust, and emotional deprivation. Similarly, the failure to resolve the theme of Care is associated with concepts like self-absorption, self-indulgence, and a sense of personal impoverishment [10]. For adolescents with diabetes, these lower levels of Hope and Care may manifest as difficulties in trusting others, experiencing social isolation, feeling entitled, suppressing emotions, and struggling with self-control. These challenges can impact how they perceive themselves, relate to others, and manage their condition effectively. Moreover, our study did not find a significant correlation between controlled HbA1c levels and the psychosocial development subscales. This result contradicts Bazzazian S et al.'s previous study, which found significant correlations between HbA1C levels and different variables, including attachment styles, psychological well-being, and quality of life [11]. However, it is essential to point out that our methodology and assessment instruments differ, which may explain the discrepancy in the results. Their study utilized the Adult Attachment Inventory (AAI) instead of PIES. Lastly, our analysis showed that adolescents living with T1DM are associated with comorbidities such as asthma, hypothyroidism, hypertension, and depression, which aligns with many published studies in the literature. In support of our findings, Fazeli Farsani S et al. conducted a study investigating the incidence of chronic comorbidities in children aged less than 19 years living with T1DM compared to a control group of children not living with T1DM. The study's findings demonstrated that children with T1DM had a statistically significantly higher prevalence of comorbidities, including cardiovascular, thyroid, pulmonary diseases, and mental disorders [12]. These findings highlight the importance of the comprehensive approach for adolescents with T1DM, taking into account not only the management of diabetes but also the identification, prevention, and treatment of associated comorbidities.

Implications and limitations

The findings of this study have profound implications for clinical practice and healthcare policies. The study highlights the critical need for a holistic approach to the care of adolescents with T1DM, encompassing not only the management of their physical health but also the recognition and support of their mental health needs. The comprehensive analysis of psychosocial development using a validated method offers valuable insights into the unique challenges faced by T1DM adolescents, enabling healthcare providers to tailor interventions and support strategies accordingly. Moreover, these findings can inform further research endeavors, fostering a deeper understanding of the psychosocial impact of T1DM and guiding the development of evidence-based interventions. In addition to clinical implications, policymakers can utilize these findings to advocate for improved access to psychological services and create supportive environments for this population, enhancing their overall well-being and quality of life. However, the present study has several limitations that warrant acknowledgment and consideration. Firstly, the study design was descriptive and cross-sectional in nature, which restricts our ability to establish causal relationships and determine the temporal sequence of events. Additionally, the inherent limitations associated with cross-sectional studies, such as recall bias and the potential for confounding variables, should be considered when interpreting the results. Secondly, the uneven sample sizes between the control group (n=220) and children living with T1DM (n=90) should be noted. This disparity in sample sizes may introduce potential biases and impact the generalizability of our findings. Thirdly, data collection occurred during the COVID-19 pandemic, which could have influenced participant responses. The pandemic's unique circumstances may have influenced the accuracy of the data collected. It is strongly recommended that future research addresses these concerns. Specifically, studies with a larger and more balanced sample size in both the control and non-control groups are needed to enhance the validity and generalizability of the findings. Moreover, conducting research during a time free from the constraints and disruptions imposed by the COVID-19 pandemic would allow for a more accurate assessment of the variables.

## Conclusions

In conclusion, this study examined the psychosocial development of adolescents with T1DM in Saudi Arabia using Erikson's stages of psychosocial development as a framework. The findings highlight the impact of T1DM on the psychosocial development of adolescents, demonstrating lower scores in certain themes compared to adolescents not living with T1DM. Contrary to some existing literature, there was no statistically significant correlation between HbA1c levels and psychosocial themes and stages. A comprehensive and holistic approach to these groups, including multidisciplinary care and access to mental health care, is needed to address the unique psychosocial challenges faced by adolescents living with T1DM and enhance their overall well-being and quality of life. Future research should aim for larger and more balanced sample sizes while also considering cultural and social influences, family dynamics, and the effectiveness of interventions. This will help optimize support for adolescents with T1DM both locally and globally.
